# Validation of the Monitoring Efficacy of Neurogenic Bowel Treatment on Response (MENTOR) Tool in a Japanese Rehabilitation Setting

**DOI:** 10.3390/jcm10050934

**Published:** 2021-03-01

**Authors:** Masashi Nomi, Atsushi Sengoku, Klaus Krogh, Anton Emmanuel, Albert Bohn Christiansen

**Affiliations:** 1 Department of Urology, Hyogo Prefectural Central Rehabilitation Hospital, Hyogo 651-2181, Japan; a_sengoku@hwc.or.jp; 2 Department of Hepatology and Gastroenterology, Aarhus University Hospital, 8000 Aarhus, Denmark; klaukrog@rm.dk; 3 GI Physiology Unit, University College Hospital, London NW1 2BU, UK; anton.emmanuel@nhs.net; 4 Medical Affairs, Coloplast A/S, 2970 Humlebaek, Denmark; dkalbc@coloplast.com

**Keywords:** neurogenic bowel, spinal cord injury, treatment assessment

## Abstract

Study design: Prospective observational study. Objective: To validate the Monitoring Efficacy of NBD Treatment On Response (MENTOR) tool in individuals with a spinal cord injury (SCI) or spina bifida, suffering from neurogenic bowel dysfunction (NBD) in a rehabilitation center in Japan. Methods: First, the MENTOR tool was translated from English to Japanese using a validated translation process. Second, the MENTOR tool was validated in a rehabilitation clinic in Japan. Participants completed the MENTOR tool prior to a consultation with an expert physician. According to the results of the tool, each participant was allocated to one of three categories regarding change in treatment: “adequately treated,” “further discussion,” and “recommended change.” The results of the MENTOR tool were compared with the treatment decision made by an expert physician, who was blinded to the results of the MENTOR tool. Results: A total of 60 participants completed the MENTOR tool. There was an acceptable concordance between individuals allocated as respectively, being adequately treated (100%) and recommended change in treatment (61%) and the physicians’ decision on treatment. The concordance was lower for individuals allocated as requiring further discussion (48%). Conclusions: In this study the MENTOR tool was successfully validated in a Japanese rehab setting. The tool will help identify individuals with SCI that need further treatment of their NBD symptoms.

## 1. Introduction

Symptoms of constipation and fecal incontinence often occur in individuals with central nervous system injury or disease [1,2]. Such symptoms are categorized as neurogenic bowel dysfunction (NBD) and have a profound negative impact on quality of life and social integration [3]. NBD is also associated with increased health service costs [4]. However, with optimal bowel management, NBD has been shown to improve [5,6,7], hence it is important to identify and treat those individuals suffering from NBD. Though several options for management of NBD exist, it has been found that NBD was a problem among 78% of individuals with spinal cord injury (SCI) and 71% had not modified any aspect of their bowel routine for more than 5 years [8]. In Japan, there are only a few specialists in NBD and guidelines on how to treat NBD were only recently published [9], making it even more difficult to identify and treat Japanese individuals who suffer from NBD [10,11].

A recently published study reviewed currently available scores for assessment of NBD in individuals with SCI [12]. However, none of these scores have yet been globally validated or accepted. The International Standards to document remaining Autonomic Function after Spinal Cord Injury (ISAFSCI) is a measure that can be used by physicians to assess the remaining autonomic function after SCI, but it is used to assess all autonomic functions and not only the bowel function [13]. The international basic bowel function data set was developed to standardize the collection of information on NBD in daily practice, but it is a static and not a dynamic measure [14]. The NBD score is a symptom-based score developed for assessment of NBD symptoms specifically in individuals with SCI [15]. Though an increase in the NBD score has been shown to correlate with a decreased quality of life, the score does not include patients’ subjective impression of their symptoms [15]. Recently, a new measure capable of reflecting change, called the Monitoring Efficacy of NBD Treatment On Response (MENTOR) tool, was developed with the objective to assess the severity of NBD in individuals with SCI by combining the NBD score with special attention symptoms (SAS), which are the elements of comorbidity that may be linked to poor bowel management [16], and patients’ perception of satisfaction with their bowel function [16]. The MENTOR tool has already been validated for use in rehabilitation clinics and gastroenterology clinics in the USA and Europe where the MENTOR tool showed good correspondence with the decisions made by expert physicians [16]. However, it has not yet been validated in Japan or any other Asian country. 

## 2. Experimental Section

In this prospective observational study, the MENTOR tool was validated in a Japanese setting. The study was approved by the Hyogo Prefectural Central Rehabilitation Hospital Ethics Committee reference number 1917.

All patients filled out an ICF prior to participating, with assistance from an onsite nurse.

### 2.1. The MENTOR Tool

The MENTOR tool consists of three components. The first component is bowel/defecation symptoms assessed by the validated NBD score. The NBD score comprises ten items which showed good reproducibility and validity, and which allow stratification into four tiers of severity, and which are significantly associated with impact on QOL [15]. Based on odds ratios for associations between items and impact on QOL, each has a corresponding number of points in the NBD score. The second component is SAS listed in Table 1. The third component is the patient’s perception of satisfaction with their bowel function which includes the following options; satisfied, acceptable, dissatisfied, and very dissatisfied.

**Figure 1:**
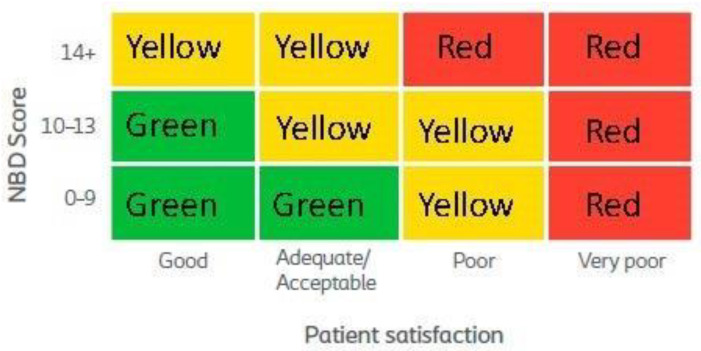
The MENTOR (Monitoring Efficacy of NBD Treatment On Response) grid to determine treatment assessment outcome. Green “Monitor”, Yellow “Discuss” and Red “Act”.

After completing all three components of the MENTOR tool, patients were assigned to one of three zones; a green, a yellow, or a red zone. As illustrated in Figure 1, the combination of an NBD score and patient satisfaction allocates the patients to one of the three zones in the MENTOR grid. Further, if an individual reports any of the listed SAS they will be moved one grid square up and to the right, effectually escalating their treatment recommendation. The green zone represents adequate treatment of individuals, the yellow zone reflects suboptimal treatment and a need of further discussions with the individual and the possibility of change in treatment and/or further monitoring and the red zone suggests inadequate treatment and a need for further examination and most likely, change in current treatment.

### 2.2. Translation of the MENTOR Tool into Japanese

To ensure that the tool was correctly translated into Japanese a thorough translation process was performed. First, the MENTOR tool was double forward translated from English to Japanese by two bilingual residents of Japan who were professionally qualified in translating. Second, the two translated versions of the tool were compared and merged into a single Japanese version. Third, a backward translation from Japanese to English was performed and this version was compared with the original English version of the MENTOR tool to identify and resolve any discrepancies between the two versions. Finally, the edited Japanese version of the MENTOR was reviewed by a panel of expert clinicians before proofreading and formatting were performed.

### 2.3. Validation of the MENTOR Tool in a Japanese Setting

The validation of the MENTOR tool was performed in one rehabilitation clinic in the Hyogo prefecture of Japan. All adults ≥18 years with a confirmed diagnosis of non-congenital SCI of more than 3 months or a confirmed diagnosis of spina bifida were eligible for inclusion if they also had a confirmed diagnosis of NBD, with use of a minimum of one method for managing their bowel function.

Of these, individuals with a scheduled consultation at the rehabilitation clinic in the period January 2020 to July 2020, were invited to participate in the study. Participants received a self-completion questionnaire comprising the MENTOR tool prior to their consultation with a physician.

To assess the ease of use of the MENTOR tool a clinician registered the time it took for each individual to complete the questionnaire and after completion each individual was asked whether the questionnaire was easy to understand (yes/no answer). Further, the clinician verified that all items of the questionnaire were completed.

After completion of the MENTOR tool, the scheduled consultation with the physician took place as per usual and the physician was not informed on the results of the MENTOR tool. At the end of the consultation, the physician registered one of the three following outcomes: (1) no treatment change, (2) discussion but no treatment change, or (3) recommendation of change in treatment due to inadequate current treatment in the physician template.

### 2.4. Statistical Analysis

All data including data from the MENTOR tool and the outcome registered by the physician were entered into a predetermined and locked Excel file. All data were analyzed using Excel including means with standard deviations for normally distributed data and proportions. The results of the MENTOR tool and the decisions made by a physician were compared by calculating the concordance of the results of the MENTOR tool with the decision made by the expert physician.

## 3. Results

A total of 57 individuals with SCI and 3 with spina bifida were included from one rehabilitation clinic located in the Hyogo prefecture, Japan. Of these, most were males (*n* = 55), and the mean age was 46.9 years (Standard deviation [SD] 14.2).

**Figure 2:**
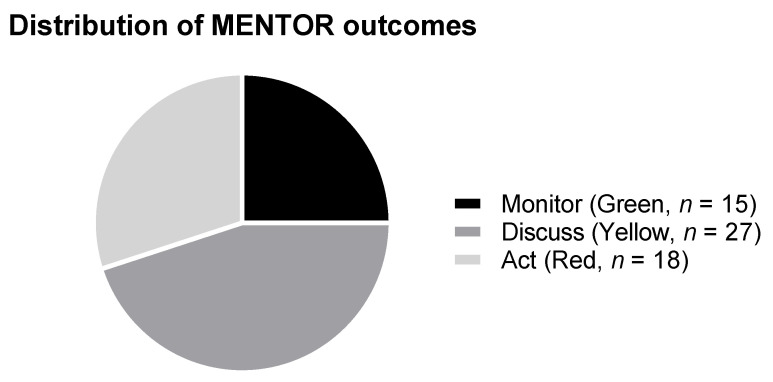
Distribution of MENTOR outcome in Hyogo Prefectural Central Rehabilitation Hospital (*n* = 60). Green “Monitor”, Yellow “Discuss,” and Red “Act”.

According to the MENTOR tool, 15 patients (25%) were allocated to the green zone indicating they received adequate treatment, 27 (45%) were allocated to the yellow zone indicating that they received suboptimal treatment, and 18 (30%) were allocated to the red zone indicating that they received inadequate treatment (Figure 2). The MENTOR tool was reported by patients to be easy to understand in 97%, and it took a mean of 4.1 min (range 1–14 min) to complete.

**Figure 3:**
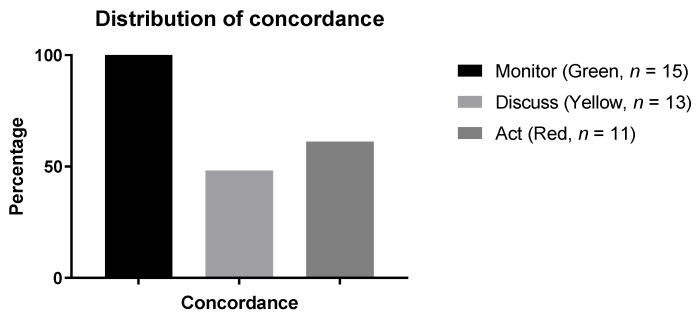
Distribution of concordance between MENTOR and clinician treatment assessment decision (*n* = 60), total concordance (*n* = 39).

When comparing the results of the MENTOR tool with the decision made by the physician, agreement was obtained in 65% of all cases (Figure 3, Table 2). There was 100% concordance with the physicians’ decision for individuals in the green zone of the MENTOR grid, 61% concordance for individuals in the red zone, and only 48% concordance for individuals in the yellow zone. Notably, of the 27 individuals in the yellow zone, 24 (89%) were not recommended change in treatment by the physician at the rehabilitation clinic.

**Figure 4:**
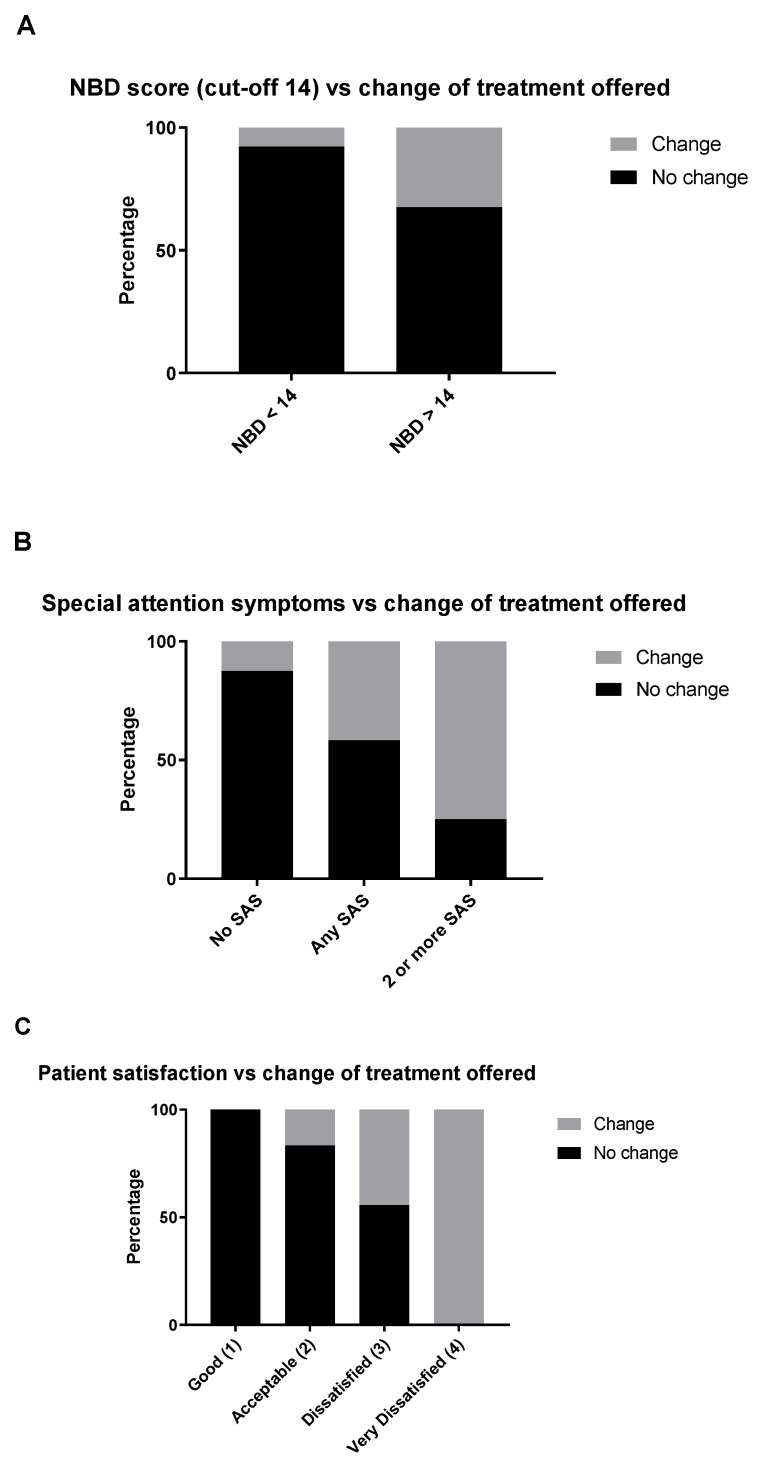
(**A**) Severe neurogenic bowel dysfunction (NBD) score associates with treatment change. (**B**) Linear association of special attention symptoms (SAS) and treatment change. (**C**) Inverse association between patient satisfaction and treatment change.

When looking at the three specific components of the MENTOR tool, we observed an association between each of the three components and recommendation of change in treatment (Figure 4). Overall, a total of 13 participants (22%) were recommended change in treatment by the physician. For the NBD score, only 2 of 26 (8%) individuals with an NBD score of less than 14 were recommended change in their treatment, while 11 of 34 (32%) individuals with an NBD score of more than 14 were recommended change in their treatment (Figure 4A). For the SAS component, 6 of 48 (13%) individuals with no SAS were recommended change in their treatment, which increased to 5 of 12 (42%) individuals with one SAS and 3 of 4 (75%) individuals with more than one SAS (Figure 4B). For the patient satisfaction component, the proportion of individuals who were recommended change in their treatment increased with dissatisfaction of their bowel function (Figure 4C). No individuals who reported that they were satisfied with their bowel function were recommended change in their treatment (0 of 14 individuals) while 6 of 36 (17%) individuals who reported their bowel function was acceptable, 4 of 9 (44%) individuals who reported they were dissatisfied with their bowel function, and 1 of 1 (100%) individual who reported he/she was very dissatisfied with the bowel function, were recommended change in their treatment.

## 4. Discussion

In this observational study the MENTOR tool was validated in a Japanese setting. We found that NBD patients’ subjective experience of treatment adequacy assessed by the MENTOR tool corresponded well to the independent decision made by the clinicians. The MENTOR tool was easy to understand and complete.

While there was acceptable concordance between individuals assigned to the green and red zone and the physicians’ decision (as shown in the combined data in Table 2), there was only 48% concordance between individuals assigned to the intermediate yellow zone and the physicians’ decision. Most of these individuals were not recommended any treatment change by the physician (24 of 27 individuals, 89%) though allocation to the yellow zone could indicate that they only had a suboptimal treatment. This could be explained by patients having a tendency not to address their symptoms at the consultation because they are unaware of the severity of their symptoms or that they are embarrassed by their symptoms [17]. Nevertheless, it is important to identify this group of patients as studies have reported that suboptimal care of bowel management has a negative impact on the quality of life [4]. Our results indicate that the MENTOR tool could help identify this group of patients.

When comparing the Japanese validation with the International validation of the MENTOR tool in rehabilitation clinics, we found an overall consistency between results e.g., the concordance of individuals allocated to respectively the green and red zone were 100% and 61% in our Japanese validation study and 86% and 68% in the international validation study [16]. This suggests that the MENTOR tool is also applicable in rehabilitation clinics in Japan. Notably, in the International validation study, the MENTOR tool was also validated by two NBD experts at two gastroenterology clinics [16]. Interestingly, there was more than 90% agreement between the results of the MENTOR tool and decisions made by the expert physicians in NBD [16]. This supports the hypothesis that the MENTOR tool is even more comparable to decisions made by the expert physicians in NBD. In countries like Japan where there are fewer experts in NBD, use of the MENTOR tool seems particularly important, as this may help identify those patients who need input from an expert in NBD.

Importantly, one study found that the severity of NBD symptoms increased significantly over time in individuals with SCI [18], which implicates that there is a need of lifelong follow-up on the severity of NBD in these patients. Indeed, the MENTOR tool would be an easy way to consistently monitor the need of further treatment of NBD. As some patients may not have follow-up visits at a gastroenterology clinic or a rehabilitation center the tool could also help physicians and caregivers in non-hospital settings to become aware of the worsening of NBD symptoms in individuals with SCI and a potential requirement of further management [19].

When patients who need further treatment of NBD are identified with the MENTOR tool, it is imperative that physicians choose the right treatment. Recently, Paralyzed Veterans of America published a clinical practice guideline for healthcare providers on how to manage NBD in adults after SCI [7]. The practical guide thoroughly describes all treatment options of NBD, indications of each treatment, and current evidence of efficacy of treatments [7]. In most countries including Japan, a stepwise approach to NBD treatment starting from the least invasive method is recommended [7,20,21]. Conservative bowel management (CBM) is first-line treatment for most patients with neurogenic bowel dysfunction. CBM includes diet and fluid management, a scheduled bowel routine, physical activity, and oral and rectal medications [7,21]. In patients with insufficient results of CBM, transanal irrigation (TAI) is most often recommended [7,21]. During TAI, feces evacuates from the bowel by introducing water into the colon and rectum through the anus [21]. If treatment with CBM and TAI fails, functional electrical stimulation of the sacral nerve or antegrade colonic irrigation either through appendicostomy or percutaneous endoscopic colostomy may be considered [7,21]. Colostomy is often considered as the last treatment option due to its invasive nature. However, colostomy is successful in a large proportion of patients and associated with a reduced bowel management time and improved quality of life [21]. Implementation of the MENTOR tool can help clinicians assess and identify when the patient should revise their current treatment following the stepwise approach.

This is the first time the MENTOR tool was translated into a non-European language. However, the NBD score, which is one of the components in the MENTOR tool, has already been translated into several languages spoken outside Europe and the USA including Japanese, Arabic, Mandarin, and Turkish [10,22,23]. In our study, 97% of participants reported that the Japanese version of the MENTOR tool was easy to understand and complete indicating that the translation of the MENTOR tool into Japanese was successful.

While it may seem paradoxical to describe the score as readily understood by patients when it contains terms like “autonomic dysreflexia” it is important to note that a key part of training of SCI patients is to help them recognize the alarm features to be aware of. As such, spinally injured individuals in a rehabilitation setting will generally have a good understanding of the features of dysreflexia.

Some limitations apply to our study. No information was given on whether specific components of the MENTOR tool e.g., the SAS, were the reason of allocation of individuals into the yellow zone. Due to lack of this information, it is not possible to explain the reason why only a few individuals in the yellow zone were recommended change in treatment by the physician. The tool was only validated in one rehabilitation clinic in the Hyogo prefecture of Japan why it may not be generalizable to other prefectures of Japan. However, a total of 60 patients participated in our study, making it the second largest study group that have validated the MENTOR tool [16].

## 5. Conclusions

We conclude that the MENTOR tool is applicable in a Japanese rehab setting. The MENTOR tool will help identify individuals with SCI who are unaware of the severity of their NBD symptoms and thereby facilitate the discussion with the physician and possibly lead to an improvement treatment.

Further studies to identify whether it can improve symptoms, reduce hospitalizations, urinary tract infections, and other comorbidities in the longer term would be interesting to pursue.

## Figures and Tables

**Table 1:** Special attention symptoms of neurogenic bowel dysfunction.

Special Attention Symptoms
Intense pain in abdomen or rectum. New or increased rectal bleeding. Hospitalization due to bowel problems. Loss of independence or change in circumstances that potentially impacts bowel care or bowel function. Episode of autonomic dysreflexia related to bowel problems.

**Table 2:** MENTOR results and agreement with physician.

	*N*	%
Total participants	60	100
Participants allocated to the three zones:		
Green zone	15	25
Yellow zone	27	45
Red zone	18	30
Concordance according to the three zones:		
Green zone	15	100
Yellow zone	13	48
Red zone	11	61
Green + Red zone	26	79
Total concordance (Green + Yellow + Red zone)	39	65
Recommendation of change in treatment in the Yellow zone: ^1^		
Yellow + Change in treatment	3	11
Yellow + No change in treatment	24	89
Total participants recommended change in treatment	11	18

^1^ Recommendation made by the physician.

## Data Availability

The data that support the findings are available from the corresponding author (M. N.) upon reasonable request.
